# Non-chemical signalling between mitochondria

**DOI:** 10.3389/fphys.2023.1268075

**Published:** 2023-09-22

**Authors:** Rhys R. Mould, Ifigeneia Kalampouka, E. Louise Thomas, Geoffrey W. Guy, Alistair V. W. Nunn, Jimmy D. Bell

**Affiliations:** ^1^ Research Centre for Optimal Health, School of Life Sciences, University of Westminster, London, United Kingdom; ^2^ The Guy Foundation, Dorset, United Kingdom

**Keywords:** biophoton, ultraweak luminescence, bystander effect, non-chemical signalling, radicals, metabolic photon emission, ultraweak photon emission

## Abstract

A wide variety of studies have reported some form of non-chemical or non-aqueous communication between physically isolated organisms, eliciting changes in cellular proliferation, morphology, and/or metabolism. The sources and mechanisms of such signalling pathways are still unknown, but have been postulated to involve vibration, volatile transmission, or light through the phenomenon of ultraweak photon emission. Here, we report non-chemical communication between isolated mitochondria from MCF7 (cancer) and MCF10A (non-cancer) cell lines. We found that mitochondria in one cuvette stressed by an electron transport chain inhibitor, antimycin, alters the respiration of mitochondria in an adjacent, but chemically and physically separate cuvette, significantly decreasing the rate of oxygen consumption compared to a control (*p* = <0.0001 in MCF7 and MCF10A mitochondria). Moreover, the changes in O_2_-consumption were dependent on the origin of mitochondria (cancer vs. non-cancer) as well as the presence of “ambient” light. Our results support the existence of non-chemical signalling between isolated mitochondria. The experimental design suggests that the non-chemical communication is light-based, although further work is needed to fully elucidate its nature.

## 1 Introduction

In 1923, Alexander Gurwitsch, in his famous onion root experiment, demonstrated that exposure of surfaces of onion root tips divided by a quartz barrier altered the incidence of mitosis (Gurwitsch, 1923). The effect was not observed when the tips were divided by opaque or glass barriers, leading Gurwitsch to speculate the source of the signal to be electromagnetic in nature and which he denominated “mitogenic radiation”. Despite such intriguing results, research into non-chemical communication (NCC) was relatively sparse until a modest renewal in the 1980s which has since continued to grow (Volodyaev and Beloussov, 2015). Despite this, there is a lack of consensus on the underlying mechanisms underpinning non-chemical communication.

One of the most controversial proposed mechanisms associated with non-chemical communication is that some form of intrinsic light is emitted and received by cells or organelles to elicit a biological response. This hypothesis is underpinned by the existence of the so-called Ultraweak Photon Emission (UPE) phenomenon, which describes the production of light from cells or organelles (distinct from delayed luminescence or bioluminescence). The spontaneous emission of photons from cells in this manner has been described by many names, including biophotons, biological auto luminescence, metabolic photon emission, as well as UPE. It arises from the production of reactive oxygen species (ROS), which oxidise nearby biomolecules, such as lipids, proteins, and DNA (Auten and Davis, 2009), forming triplet excited carbonyl species. Energy transfer from these intermediates to nearby pigments or molecular oxygen results in the formation of excited pigments and singlet oxygen, respectively. Relaxation to the ground state in each of these species yields a photon (Pospíšil et al., 2014). The intensity of emission is reported to be extraordinarily low, in the range of 1–1,000 photons cm^-2^ s^-1^ and range from around 200–800 nm. As mitochondria are a major source of ROS, they are in turn considered a primary or major source of UPE (Van Wijk et al., 2020). Within a cell, UPE may facilitate energy transfer from the site of origin to surrounding aromatic structures, such as microtubules (Ilan, 2022), which then propagate signals throughout the cell. Such a pathway, driven by elevated ROS levels, have been modelled and proposed to contribute to the development of disease states such as Alzheimer’s Disease (Kurian et al., 2017). As mitochondrial dynamics, such as mitophagy, fusion, and fission, play a major role in important role in neurological function (Yang et al., 2021), there is also a substantial body of literature describing potential roles of UPE within the brain, including an observed coupling between recoded photon emission and the brain’s alpha rhythm (Wijk et al., 2008). It has also been proposed that nerve fibres may transmit cell-based UPE throughout neural circuits and contribute to neural signal transmission (Tang and Dai, 2014). UPE may also have clinical or diagnostic applications and has been used to distinguish between malignant and non-malignant cells *in vitro* and *in vivo* (Murugan et al., 2020).

Numerous publications describing non-chemical communication attribute their observations to UPE. Notable examples include alterations in the bioenergetics of isolated rat mitochondria (Bat’ianov, 1984), activation of chemically separated neutrophils (Shen et al., 1994), modulation of ATP and intracellular Ca^2+^ between isolated neuroblastoma and sensory neurons (Chaban et al., 2013), and changes in cellular structure and total protein content between Caco-2 intestinal epithelial cells (Farhadi et al., 2007). A review by Scholkmann et al. (2013) provides a comprehensive publication history of developments in this area of research.

Light-cell interactions are well established, ranging from ultraviolet-based damage to physiological responses in cells following red to near-infrared-light exposure utilised in the field of photobiomodulation (formally known as low level laser therapy). Despite this, there is significant debate on the feasibility of UPE as a means of non-chemical communication. The primary counter argument is that the extremely low reported intensity of UPE makes it unlikely that a potential receptor can detect UPE as a “signal” above the inherent “noise” in a cell (Kučera and Cifra, 2013). Furthermore, whilst the observations themselves are considered valid, there is often a failure to account or control for other potential mechanisms, such as vibrational or volatile-based communication. One such example described a significant induction of cell death through a non-aqueous pathway attributed to UPE (Potapovich and Kostyuk, 2021), but further investigation by this lab revealed that the observed effect was dependent on solvent volatility, leading to the conclusion that the effect was likely a result of volatile communication rather than a light-based pathway (Richard Mould et al., 2022).

The re-emergence of such proposed mechanisms, as well as the pre-existing literature, has sparked a resurgence into non-chemical signalling (Volodyaev and Beloussov, 2015). In this paper, we investigate non-chemical communication between chemically and physically isolated mitochondria from cancer and non-cancer cells.

## 2 Methods

### 2.1 Cell culture

MCF7 cells (ATCC, United Kingdom) were grown in MEM (ThermoFisher, United Kingdom) supplemented with 10% FBS (ThermoFisher, United Kingdom), 1% l-glutamine, and 1% penicillin/streptomycin (Merck, United Kingdom). MCF10A cells (ATCC, United Kingdom) were grown in DMEM:F12 media supplemented with 5% Horse serum (ThermoFisher, United Kingdom), 20 ng/mL epidermal growth factor (Merck, United Kingdom), 0.5 μg/mL hydrocortisone (Merck, United Kingdom), 100 ng/mL cholera toxin (Merck, United Kingdom), 10 μg/mL insulin (Merck, United Kingdom), and 1% penicillin/streptomycin. Cells were maintained in a humidified 37°C 5% CO_2_ environment.

### 2.2 Mitochondrial isolation

Mitochondria were isolated from whole cells using the Mitochondria Isolation Kit for Cultured Cells (Abcam, United Kingdom). Briefly, cells were harvested and centrifuged at 1000 RCF for 5 min. Pellets were frozen at −80°C overnight to weaken membranes. Cells were defrosted, resuspended to 5 mg/mL in Kit Reagent A, and kept on ice for 10 min. Cells were then homogenized with 30 strokes of a Dounce homogenizer with pestle B, and then centrifuged at 1000 RCF for 10 min at 4°C. Supernatant was set aside, and the pellet resuspended in Kit Reagent B, homogenized with 30 strokes of a Dounce homogenizer with pestle B, and then centrifuged at 1000 RCF for 10 min. The supernatants were combined and centrifuged at 12,000 RCF for 15 min. The supernatant was discarded, and the pellet resuspended in 500 µL Kit Reagent C. Isolated mitochondria were quantified using a Bradford Assay, and their presence confirmed through staining with MitoTracker Deep Red with visual inspection under a fluorescence microscope (See Supplementary Figure S1).

### 2.3 Mitochondrial Assay Buffer

Following isolation, mitochondria were resuspended and tested in Mitochondrial Assay Buffer (MAB) prepared as follows: 110 mM mannitol, 70 mM sucrose, 10 mM KH_2_PO_4_, 5 mM MgCl_2_, 2 mM HEPES, 1 mM EGTA, 0.2% weight/volume BSA (all Merck, United Kingdom) in ultrapure water (Rogers et al., 2011).

### 2.4 Production of ROS in isolated mitochondria

The production of ROS in isolated mitochondrial samples was tested using the MitoSOX Stain (ThermoFisher, United Kingdom). Isolated mitochondria were added to a black-walled 96 well plate, spiked with MitoSOX Red (final concentration = 5 µM) and incubated for 15 min in the dark at 37°C 5% CO_2_. Changes in MitoSOX fluorescence (excitation 510/emission 580 nm) were measured using the BMG Labtech NovoStar plate reader. A baseline was measured for 16 s, at which point antimycin (final concentration 244 µM) or MAB was injected into appropriate wells. Fluorescence was then measured for a further 234 s (total time = 250 s).

### 2.5 Non-Chemical Communication Assay

**FIGURE 1:**
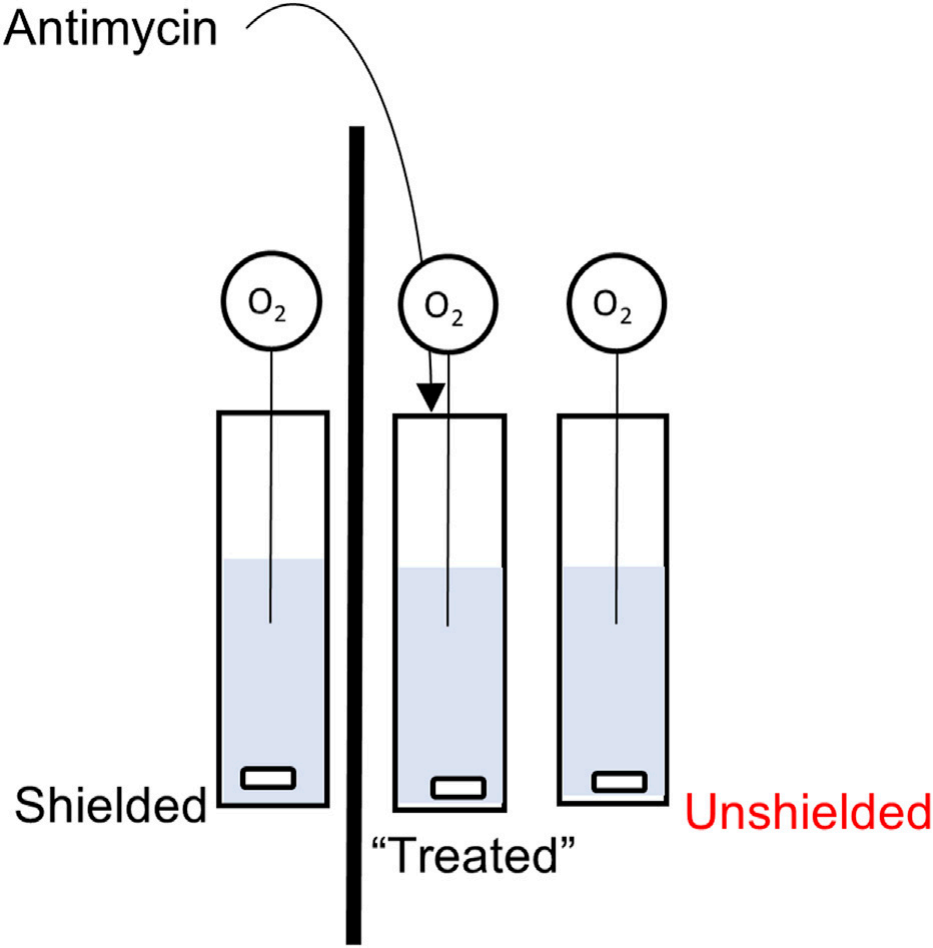
Schematic of the Non-Chemical Communication Assay experimental setup.

To investigate potential non-chemical/non aqueous signalling between mitochondria, a bespoke non-chemical signalling assay was designed (See Figure 1). The principle of the assay was to induce oxidative stress in one population of mitochondria, and then to measure the response, in terms of mitochondrial respiration, in populations of chemically separated mitochondria. Isolated MCF7 or MCF10A mitochondria were divided equally between three quartz cuvettes (Helma, Ger). The final volume in each cuvette was 2 mL, consisting of 166 µL isolated mitochondria in 1,834 µL Mitochondrial Assay Buffer (MAB). Each cuvette contained a magnetic stirrer bar and was capped with parafilm to inhibit any potential chemical transmission between cuvettes. The cuvettes were arranged in a row, window to window, with one pair separated by a light-absorbing opaque barrier made from aluminium foil. The central cuvette would receive treatment with antimycin. The cuvette separated by the barrier is referred to as “Shielded” and the third cuvette had no such barrier as is referred to as “Unshielded”. Oxygen consumption in each cuvette was measured with a FireSting O_2_ meter (PyroScience, Ger), with individual needle-like probes and a separate temperature probe to compensate for changes in temperature. Oxygen consumption was measured for 120s, at which point 50 µL 10 mM antimycin was injected via Hamilton syringe into the central cuvette (final concentration 244 µM). Oxygen consumption was then simultaneously measured for a further 130s (total measurement time of 250s) in each cuvette. Experiments were performed either exposed to “ambient” light conditions in the laboratory, or within a light-proof box. These conditions are referred to as “light” and “dark” respectively.

### 2.6 Statistics

Differences in the rate of mitochondrial oxygen consumption were analysed using a mixed linear effects model written in R, version 3.6.1 (R Core Team (2019) using the package “nlme”. Pinheiro J, Bates D, DebRoy S, Sarkar D, R Core Team (2020) (https://CRAN.R-project.org/package=nlme). Graphs were generated with GraphPad Prism (United States) version 8.2.0.

## 3 Results

### 3.1 NCC in isolated mitochondria

**FIGURE 2:**
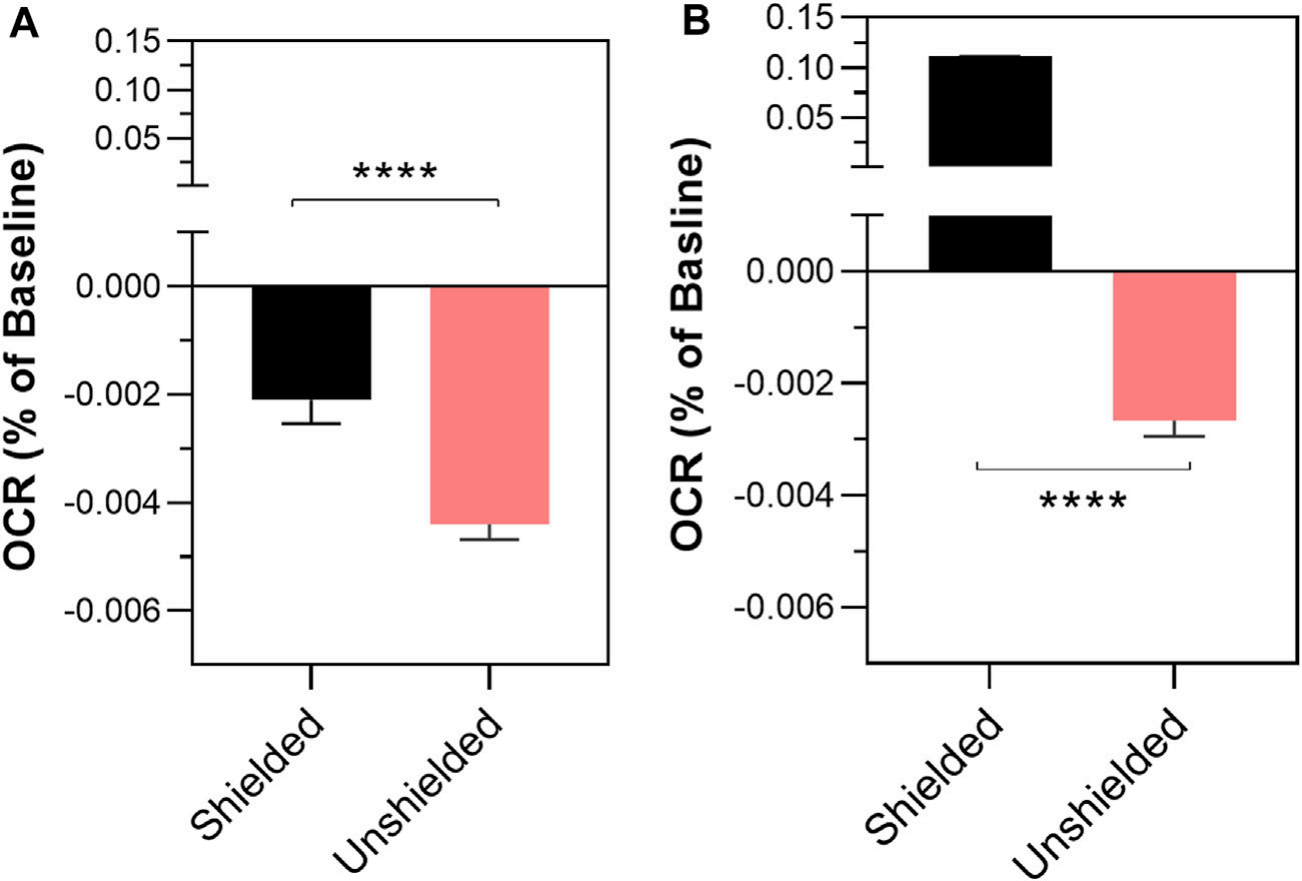
The effect of non-chemical communication on mitochondrial function over time between chemically separate populations of isolated MCF7 mitochondria **(A)** and MCF10A mitochondria **(B)**. Mitochondrial function assessed by measurement of O_2_ over time. Data presented as the mean change in oxygen consumption over time (Oxygen Consumption Rate, OCR) as a percentage of the baseline period ±SE. Differences between unshielded and shielded mitochondria analysed via Mixed Linear Effects model, considered significant when *p* < 0.05. ****: *p* < 0.0001. *n* = 12.

Induction of oxidative stress following the antimycin injection (confirmed by mitochondrial ROS assay, see Supplementary Figure S2) into the treatment cuvette caused a significant decrease in the oxygen consumption rate (OCR) in the unshielded-cuvette compared to the shielded-cuvette in both MCF7 (−0.004% per sec ±0.0003% vs. −0.021% per sec ±0.0004, *p* < 0.0001) and MCF10A mitochondria (−0.003% per sec ±0.0003% vs. 0.1109% per sec ±0.0004, *p* <0.0001) (Figure 2). To ensure that changes in OCR were caused by mitochondria, a control experiment was carried out in which the unshielded and shielded cuvettes contained MAB alone, with no mitochondria. In this experiment (Supplementary Figure S3), there was no significant difference between the unshielded and shielded mitochondrial response.

### 3.2 NCC in cancer vs. non-cancer cell lines

**FIGURE 3:**
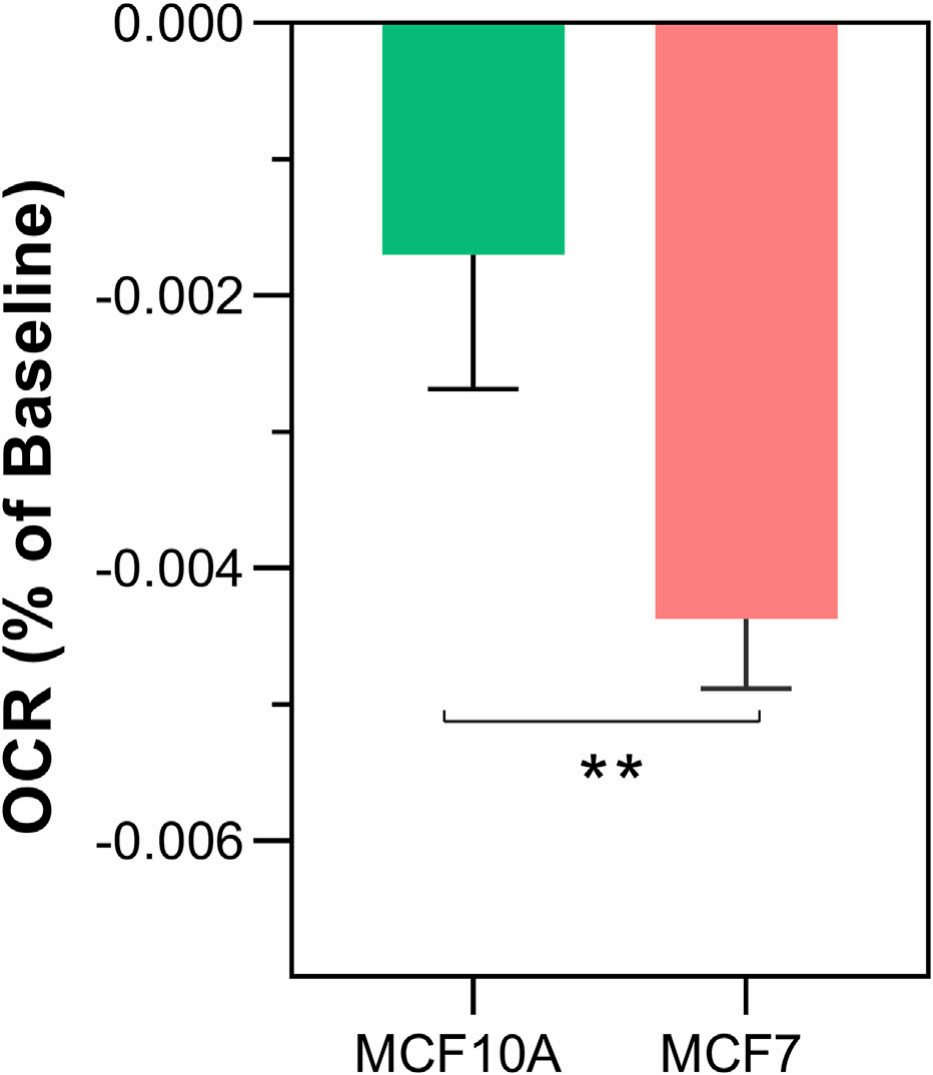
Differences in non-chemical communication on mitochondrial function between MCF7 mitochondria and MCF10A mitochondria. Mitochondrial function assessed by measurement of O_2_ over time. Data presented as the mean change in oxygen consumption over time (Oxygen Consumption Rate, OCR) as a percentage of the baseline period ±SE. Differences between unshielded and shielded mitochondria analysed via Mixed Linear Effects model, considered significant when *p* < 0.05. ****: *p* < 0.0001. *n* = 12.

Once a reproducible protocol was established for the detection of non-chemical communication between isolated mitochondria through changes in OCR, we compared potential differences between cancer and non-cancer cells (Figure 3 **)**. The results show that the rate of OCR change was significantly greater in MCF7 (cancer) compared to MCF10A (non-cancer) cells lines (−0.00426% ± 0.0007 per sec vs. −0.00201% per sec ±0.0004, *p* < 0.0044).

### 3.3 Effect of ambient room light on UPE production

**FIGURE 4:**
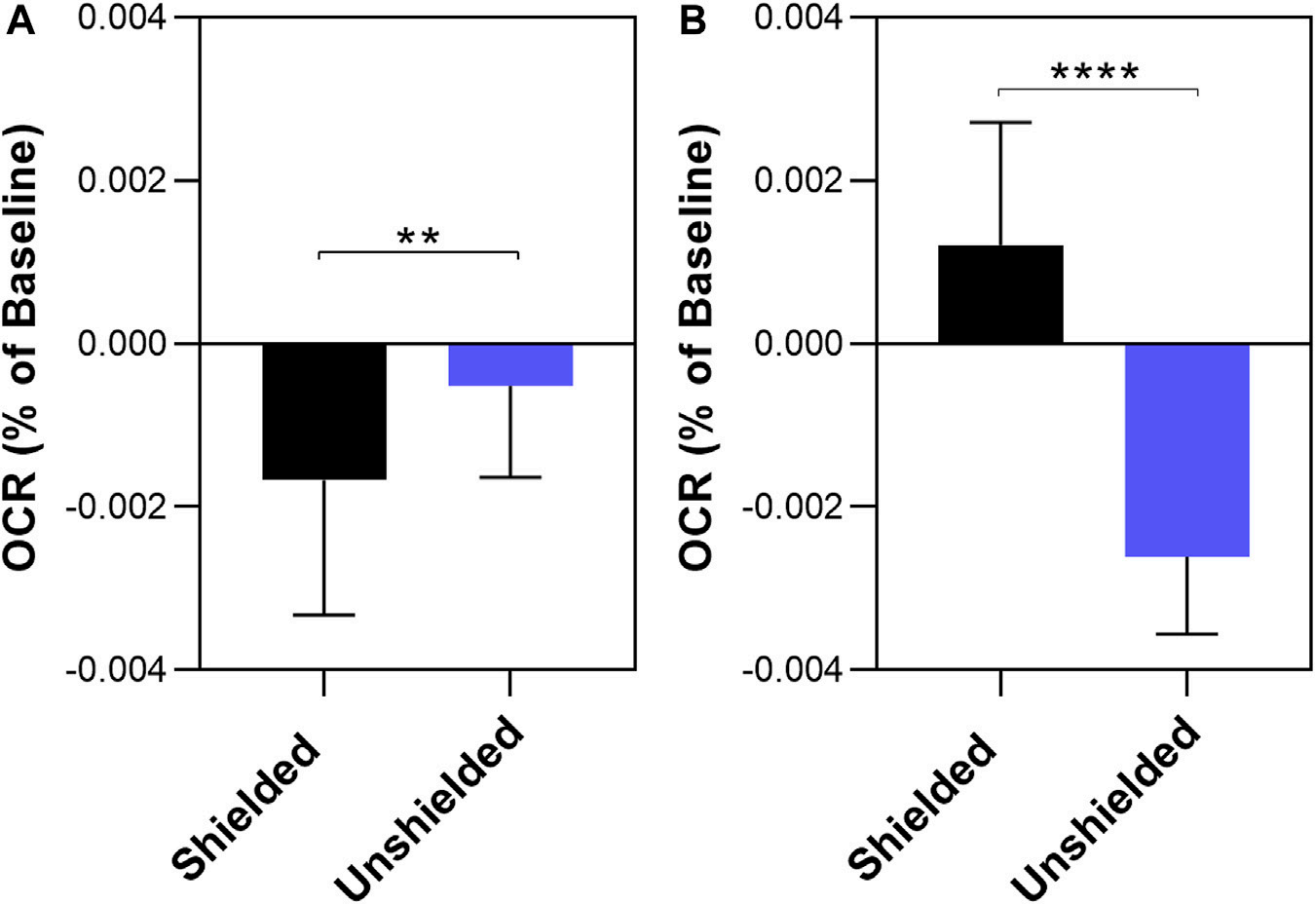
The effect of non-chemical communication on mitochondrial function over time between chemically separate populations of isolated MCF7 mitochondria **(A)** and MCF10A mitochondria **(B)**. Mitochondrial function assessed by measurement of O_2_ over time. Data presented as the mean change in oxygen consumption over time (Oxygen Consumption Rate, OCR) as a percentage of the baseline period ±SE. Differences between unshielded and shielded mitochondria analysed via Mixed Linear Effects model, considered significant when *p* < 0.05. ****: *p* < 0.0001. *n* = 12.

**FIGURE 5:**
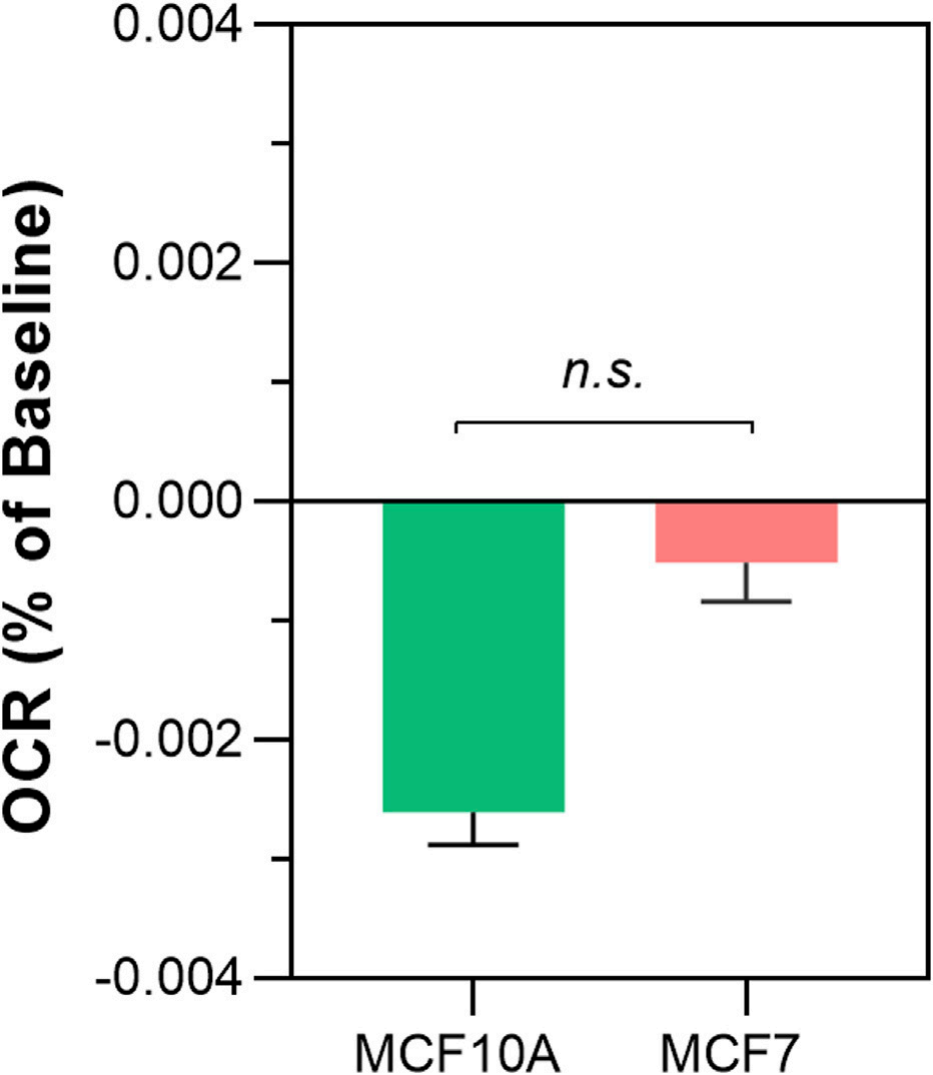
Differences in non-chemical communication on mitochondrial function between MCF7 mitochondria and MCF10A mitochondria in dark conditions. Mitochondrial function assessed by measurement of O_2_ over time. Data presented as the mean change in oxygen consumption over time (Oxygen Consumption Rate, OCR) as a percentage of the baseline period ±SE. Differences between unshielded and shielded mitochondria analysed via Mixed Linear Effects model, considered significant when *p* < 0.05. ****: *p* < 0.0001. *n* = 12.

To determine if ambient room light influenced the results reported in Figure 2 and Figure 3, we repeated the above experiments screened from any ambient light, referred to as “dark” conditions. The results are shown in Figure 4. Again, a significant decrease in OCR was observed in the shielded MCF7 mitochondria compared to the unshielded mitochondria (−0.00167% ± 0.0003% per sec vs. −0.00052% ± 0.0005% per sec, *p* = 0.0159). However, in isolated MCF10A mitochondria OCR was increased in the unshielded cuvette compared to the shielded cuvette (−0.00261% ± 0.0003% per sec vs. 0.0012% ± 0.0004% per sec, *p* <0.0001). As before, we compared changes in OCR between the two cell lines (Figure 5). There was no significant difference in the responses of MCF10A mitochondria and MCF7 mitochondria (−0.00261% ± 0.0003% per sec 0.00052% ± 0.0003% per sec, *p* = 0.091).

### 3.4 Comparing “light” and “dark” experiments

**FIGURE 6:**
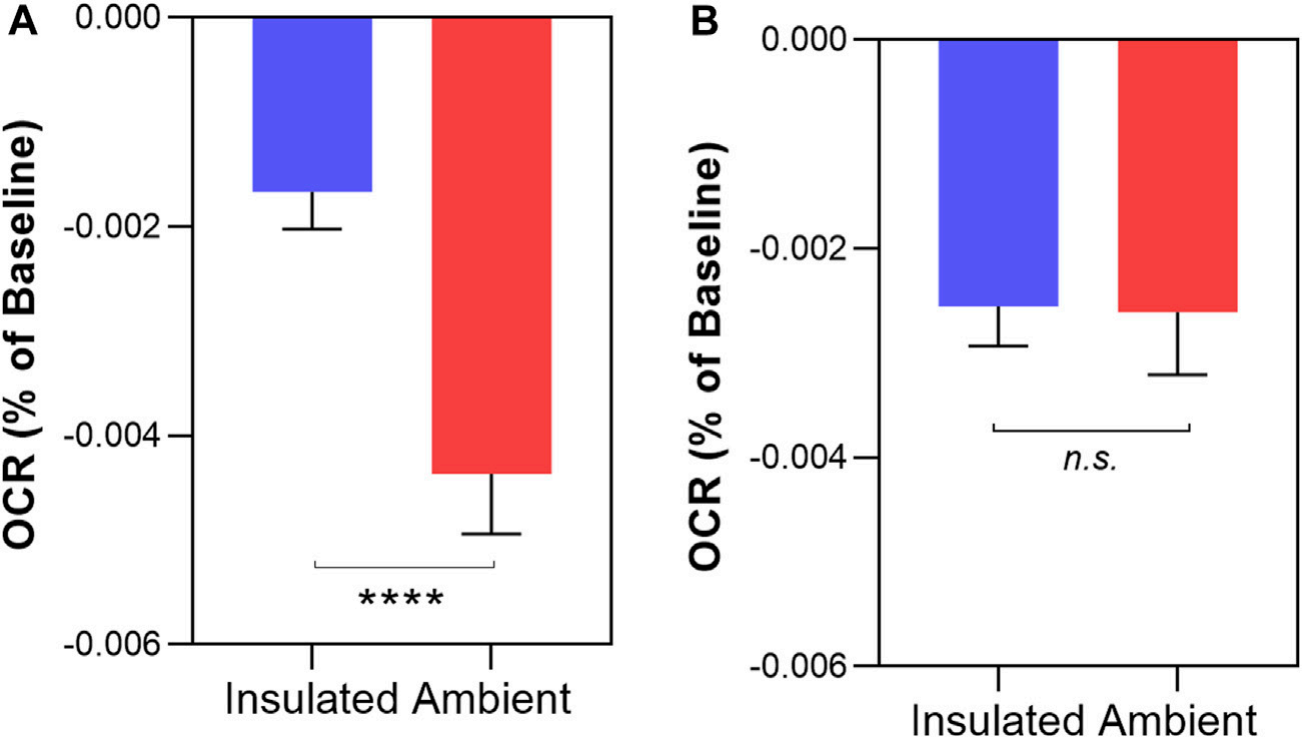
Comparing non-chemical communication in dark and light conditions in isolated MCF7 mitochondria **(A)** and MCF10A mitochondria **(B)** Mitochondrial function assessed by measurement of O_2_ over time. Data presented as the mean change in oxygen consumption over time (Oxygen Consumption Rate, OCR) as a percentage of the baseline period ±SE. Differences between unshielded and shielded mitochondria analysed via Mixed Linear Effects model, considered significant when *p* < 0.05. ****: *p* < 0.0001 *n.s*: not significant. *n* = 12.

Finally, we compared the change in OCR between unshielded mitochondria in light conditions *versus* dark conditions, shown in Figure 6. In MCF7, the rate of OCR change was significantly higher in light conditions compared to dark conditions (−0.00437% ± 0.0006 per sec vs. −0.00167% ± 0.0003 per sec, *p* < 0.0001). In MCF10A mitochondria, there was no significant difference in the rates of OCR change between light and dark conditions (−0.00261% ± 0.0003% vs. −0.00255% ± 0.0006, *p* = 0.92).

## 4 Discussion

In this paper we present evidence that validates and extends the role of non-chemical communication in biological systems, principally photonic in nature. The underlying plausibility comes from both evidence and theory that biological metabolism can produce photons, especially during oxidative stress, and that the cell is packed with photon accepting molecules. For instance, aromatics and those containing double bonds. These range from aromatic amino acids, to nucleotides, to flavins, to iron containing cytochromes, to quinones, to unsaturated fatty acids and cholesterol, as well as key electron carriers like NAD(P)H.

Interactions between light and cellular matter are well documented. For instance, the transduction of light into an electrical signal by photoreceptors in the eye constitutes vision (Ebrey and Koutalos, 2001), while ultra-violet (UV) light triggers melanocytes to produce melanin, and at a molecular level can damage DNA (Rastogi et al., 2010). At longer wavelengths, light may have beneficial effects, including improved mitochondrial function (Silveira et al., 2019; Amaroli et al., 2021; Pope and Denton, 2023). In the field of photobiomodulation (PBM), longer wavelength light exposure to skin has been touted as a treatment for a wide range of inflammation-based conditions (Demidova-Rice et al., 2012; de Freitas and Hamblin, 2016).

One of the earliest indications that life could be utilising photonic homeostasis was shown by Alexander Gurwitsch in his famous onion experiment where so called “mitogenetic signals” could pass through a quartz plate, but not a glass one (Gurwitsch, 1923). This did indeed suggest that the signal was light based, and probably UV. The original idea arose from the scientific zeitgeist of the time that life was very much an electromagnetic phenomenon (Volodyaev and Beloussov, 2015). In time, this led to many experimental studies to verify this effect, including using isolated mitochondria separated by a quartz partition (Bat’ianov, 1984), on which we have based our experiments.

To verify this effect, we stimulated the production of UPE through the induction of oxidative stress in a population of isolated mitochondria and monitored changes in mitochondrial function (through changes in oxygen consumption) in a second, chemically isolated population, compared to a third population that was shielded from the others with an opaque barrier. After establishing that our treatment indeed increased ROS production in isolated mitochondria, we found that at ambient laboratory light conditions, the rate of O_2_ consumption was significantly decreased in the unshielded MCF7 and MCF10A mitochondria compared to their shielded counterparts. The change in O_2_ consumption was observed in both “light” and “dark” experimental conditions. Interestingly, there was a significant difference between the “light” and “dark” unshielded MCF7 mitochondria suggesting some interaction between ambient room light and these mitochondria. More work would be needed to investigate the nature of this observation. Each of the untreated cuvettes, shielded and unshielded, were identical in their contents and received no treatment. Thus, this suggests any observed change between cuvettes is a consequence of a signal emitted from the treated cuvette. This would suggest the transmission of a signal that can modulate mitochondrial function at a distance without the need for direct chemical contact–a non-chemical communication pathway. As the only difference between each cuvette was the presence of an opaque barrier, it is plausible that this pathway could be mediated by light–UPE.

We also found that there was a significant difference in the response of mitochondria obtained from cancerous and noncancerous lines, with the mitochondria from cancer cells exhibiting a larger effect size. This would suggest either a stronger signal emitted from the treated mitochondria, or higher sensitivity to signals in the receiving mitochondria. In either case, these findings align with general observations of mitochondrial reprogramming in cancer cells, namely, elevated ROS which confer a proliferative advantage, which is usually associated with the Warburg effect and thus a shift to aerobic glycolysis (Schumacker, 2006).

As described above, interactions between the mitochondria and light across the spectrum are well described. Incidence of red to near infra-red (approximately 800–2,500 nm) is associated with increased ATP production (Tafur and Mills, 2008) and increased ROS production (Pooam et al., 2021). Chromophores thought to be responsible for the signal transduction at these wavelengths include cytochrome *c* (Hamblin, 2018)*.* Shorter wavelengths, between blue and green (∼400–540 nm) have been shown to inhibit proliferation through changes in calcium and ROS through the activation the calcium channel TRPV1 (Wang et al., 2017). The effects of UV light (defined as 100–400 nm) on mitochondria are also well defined - chiefly a deleterious increase in ROS because of DNA damage, to which mitochondrial DNA, lacking the DNA damage repair pathways of the nuclear counterparts, are particularly sensitive to Brand et al. (2018).

With such interactions and effects established, we reason that UPE elicits similar effects, albeit at a lower magnitude, because of the reported lower intensity. UPE is reported to span UV-C to NIR (Cifra and Pospíšil, 2014). In our results, we observe a decrease in oxygen consumption rate in “unshielded” mitochondria compared to the shielded mitochondria, interpreted as a reduction in mitochondrial function. This corroborates with literature describing lower wavelength photons decreasing function as described above.

Purported NCC effects involving light have been critiqued in the literature under two chief arguments; that the low intensity of UPE makes it unlikely to produce significant biological effect, and that experimental design in such studies often fail to account or control for alternative mechanisms (Kučera and Cifra, 2013). In this work, we have described a significant and robust NCC effect between isolated mitochondria. Numerous aspects of our experiential design control for competing alternative mechanisms strongly point to UPE as the responsible mechanism. The capping of individual cuvettes reduces the likelihood of volatile communication. We know that UPE is tied to ROS elevation, and we observe the effect only following the induction of ROS with antimycin. The use of quartz cuvettes allows for the transmission of light across the spectrum. Finally, the effect is not observed when the cuvettes are divided by an opaque barrier. Thus, we argue that light, in the form of UPE, is the most likely candidate for this effect.

Our data does seem to support the fact that indeed, the two cell types do have different metabolic profiles. MCFA10A cells are reliant on oxidative phosphorylation and generally display highly connected mitochondrial networks, while the MCF7s seem to be more glycolytic with more fragmented mitochondria (Supplementary Figure S3). PBM “mito-tuning” could have therapeutic potential.

The potential for light-mediated communication within a cell or organism has significant implications for how we consider homeostasis. In effect, there is a whole relatively unexplored aspect to biology beyond traditional biochemistry and pharmacology that utilises fields and light, which utilises biological coherence and obeys the laws of dissipative self-organising far from equilibrium structures. This may extend even to the mitochondria itself, which may support quantum effects such as tunnelling and coherence (Nunn et al., 2016). These ideas have been muted by many scientific giants, ranging from Gurtwitsch, Schrödinger, Prigogine, Szent-Gyorgyi, Fröhlich, to Chance to Popp and more recently, Levin. For example, a fascinating proposal is for nearby structures, such as the tubulin, which seems to contain a network of specifically placed chromophores, such as tryptophan that make up part of the cell’s cytoskeleton, act as “fibre optic” network to propagate energy around the cell via a process called super-radiance. Such a mechanism has been simulated in a model of Alzheimer’s Disease, in which increased ROS-based UPE result in the restructuring of the cell’s cytoskeleton (Kurian et al., 2017). Furthermore, UPE itself may provide new opportunities to study mitochondrial function in a non-invasive manner, in turn contributing to the roles of mitochondria in disease, aging, or as a potential diagnostic tool (Van Wijk et al., 2020).

### 4.1 Limitations

Our chief limitation in this study is the lack of direct evidence of the production of light. It is therefore of upmost importance that future work centres around the detection and characterisation of UPE from cellular and/or mitochondrial samples. We would note here that although there are several studies reporting the spontaneous emission of light from samples, there are questions as to whether they are truly detecting UPE, or photons from the separate phenomenon of delayed luminescence, also known as the long lasting after glow (Scordino et al., 2014).

Delayed luminescence itself also is an additional separate limitation of our study. As a mechanistically distinct source of photons, we are unable to distinguish whether the proposed light-based effects are a result of delayed luminescence photons, or UPE photons, and indeed it has been suggested that there is a relationship between mitochondrial status and delayed luminescence intensity (Tian et al., 2022).

## 5 Conclusion

The phenomenon of UPE, in our view, warrants increased interest and study. The advancement and improvement of light-detecting technologies, such as photomultiplier tubes, means that the detection of photons is now feasible, despite the purported low intensities. Such research, combined with the work determining the roles of UPE, may change how we think about inter-cell signalling. One of the key things we will need to discover is the “photonic language”, so we can learn how to retune the system when it goes wrong. This will probably require understanding both the intensity/dose, and the spectral combinations and temporality required to modulate this system. Many groups are already learning how to do this with the emerging field of photobiomodulation around tuning mitochondrial function.

## Data Availability

The raw data supporting the conclusion of this article will be made available by the authors, without undue reservation.

## Supplementary material

SUPPLEMENTARY FIGURE S1 Typical brightfield and fluorescent microscopy images of assay buffer or isolated MCF7 mitochondria, in the presence or absence of MitoTracker Deep Red. **(A)**: Brightfield image of buffer alone in the absence of MitoTracker. **(B)**: Brightfield image of isolated mitochondria in in the absence of MitoTracker. **(C)**: Fluorescent image of isolated mitochondria stained with MitoTracker Deep Red. **(D)**: Fluorescent image of assay buffer stained with MitoTracker DeepRed.

SUPPLEMENTARY FIGURE S2 The effect of antimycin injection on mtROS in MCF7 Mitochondria **(A)** and MCF10A Mitochondria **(B)**. MtROS measured using the MitoSOX assay. Data presented as mean ± SD (shaded area) fluorescent intensity expressed as percentage of the baseline period. Differences in response analysed by student’s *t*-test on the area under each curve, considered significant when *p* < 0.05. ****: *p* < 0.0001. *n* = 3.

SUPPLEMENTARY FIGURE S3 The effect of treating isolated MCF7 mitochondria on chemically isolated cuvettes containing buffer alone. Mitochondrial function assessed by measurement of O_2_ over time. Data presented as the mean change in oxygen consumption over time (Oxygen Consumption Rate, OCR) as a percentage of the baseline period ± SE. Differences between unshielded and shielded mitochondria analysed via Mixed Linear Effects model, considered significant when *p* < 0.05. n.s: not significant. *n* = 3.

SUPPLEMENTARY FIGURE S4 Mitochondrial morphology of MCF7 **(A)** and MCF10A **(B)** cells. Mitochondria stained with MitoTracker Deep Red and imaged using EVOS FL2 fluorescent microscope using the EVOS 60X, fluorite, LWD, 0.75NA/1.28WD objective.
