# Supplementary figures and images for: Non-chemical signalling between mitochondria
Source: Front Physiol. 2023 Sep 22;14:1268075. doi: 10.3389/fphys.2023.1268075 (PMC10560087; doi:10.3389/fphys.2023.1268075)

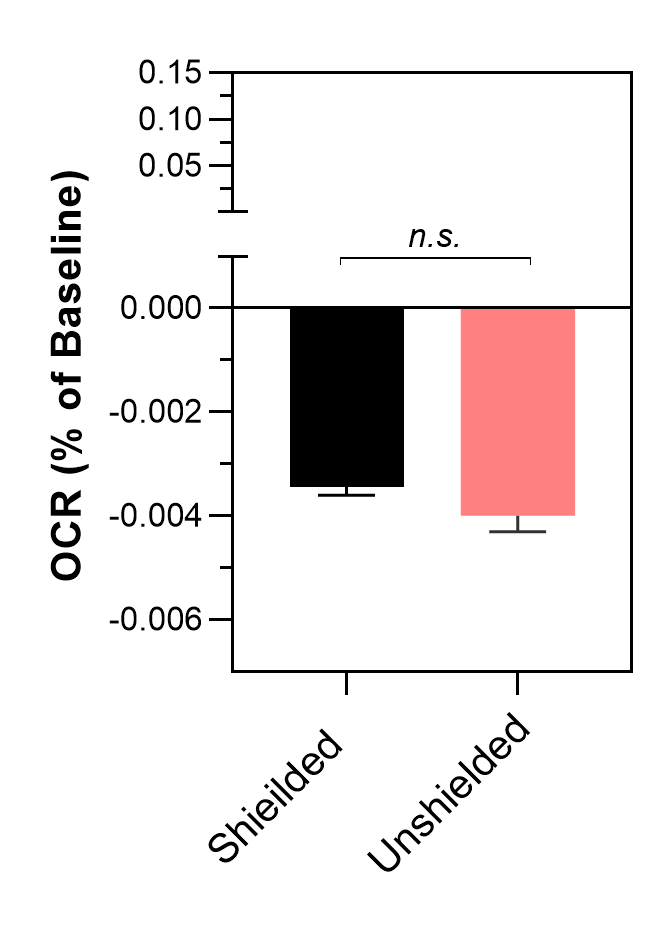

Supplement: Supplementary file 1 [file Image3.tif]

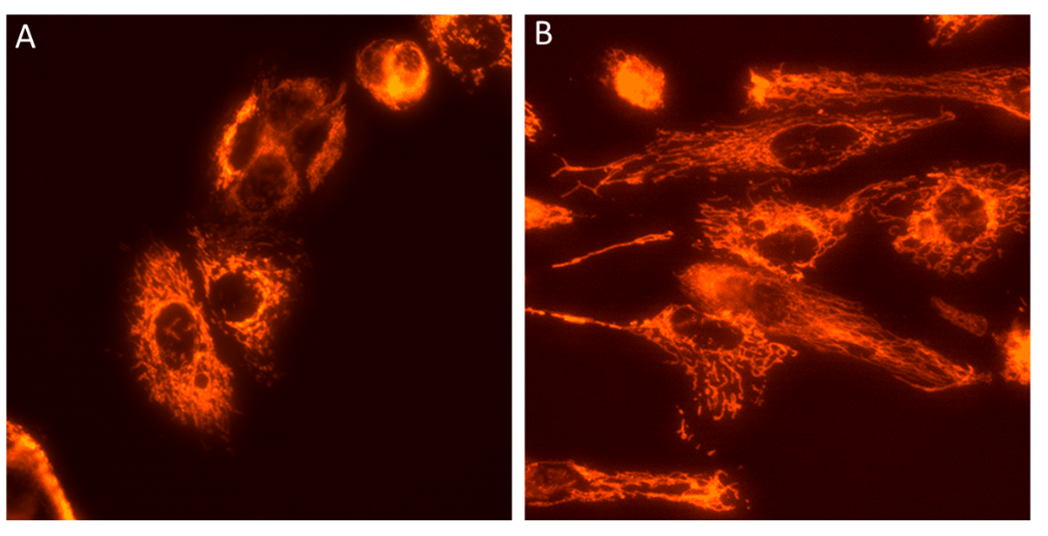

Supplement: Supplementary file 2 [file Image4.tif]

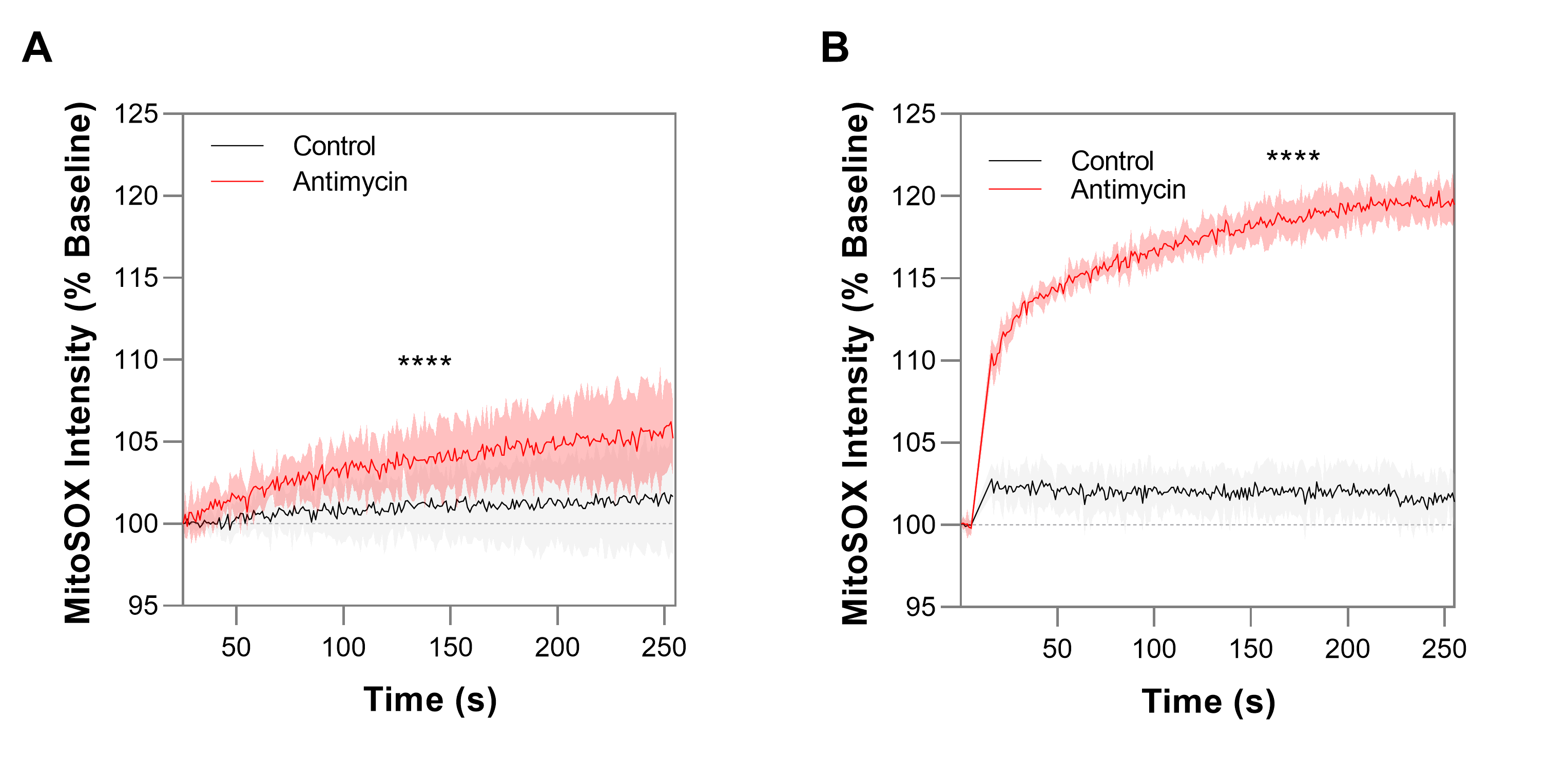

Supplement: Supplementary file 3 [file Image2.tif]

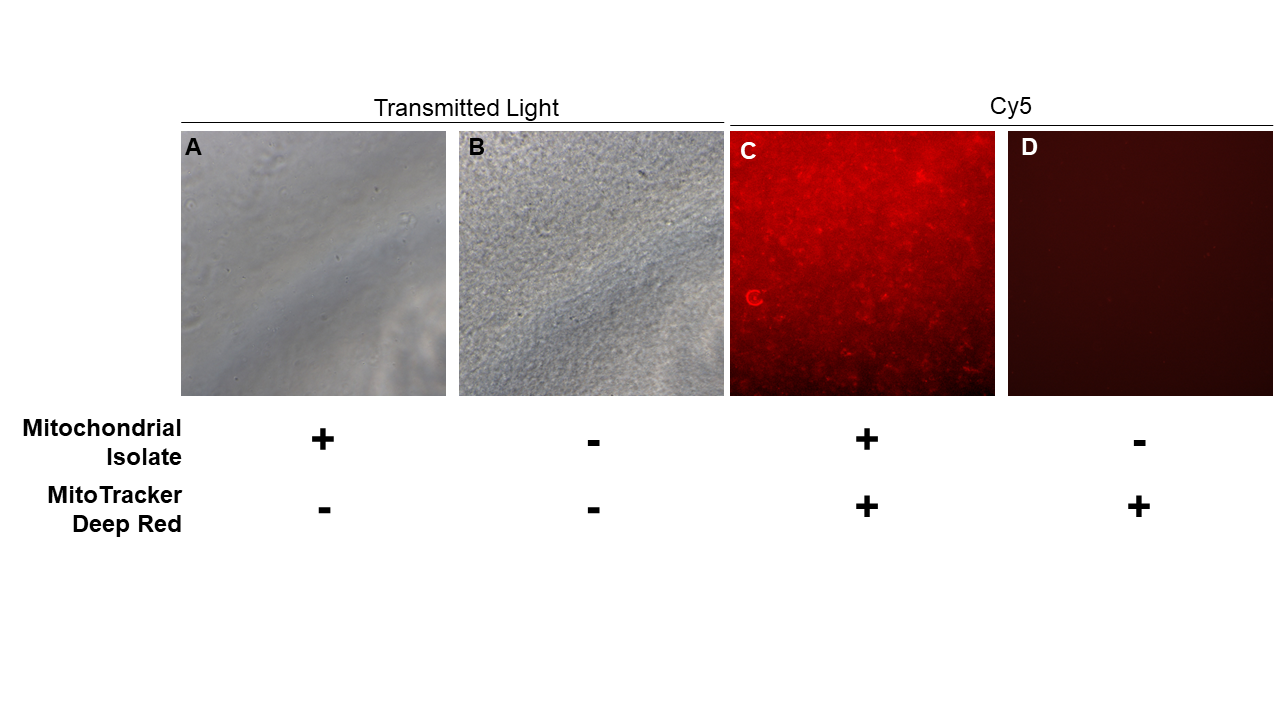

Supplement: Supplementary file 4 [file Image1.tif]
